# Efficacy of bifenthrin-impregnated bednets against *Anopheles funestus *and pyrethroid-resistant *Anopheles gambiae *in North Cameroon

**DOI:** 10.1186/1475-2875-5-77

**Published:** 2006-09-11

**Authors:** Mouhamadou Chouaibou, Frédéric Simard, Fabrice Chandre, Josiane Etang, Frédéric Darriet, Jean-Marc Hougard

**Affiliations:** 1 Organisation de Coordination pour la lutte contre les Endémies en Afrique Centrale (OCEAC), Yaoundé, Cameroun; 2 Institut de Recherche pour le Développement (IRD), UR016, Montpellier, France; 3 Université de Yaoundé I, Yaoundé, Cameroun; 4 Institute of Medical Research and Studies of Medicinal Plants (IMPM), Ministry of Scientific Research and Innovation, Yaoundé, Cameroun

## Abstract

**Background:**

Recent field studies indicated that insecticide-treated bednets (ITNs) maintain their efficacy despite a high frequency of the knock-down resistance (*kdr*) gene in *Anopheles gambiae *populations. It was essential to evaluate ITNs efficacy in areas with metabolic-based resistance.

**Methods:**

Bifenthrin was used in this experiment because it is considered a promising candidate for bednets impregnation. Nets were treated at 50 mg/m^2^, a dose that has high insecticidal activity on *kdr *mosquitoes and at 5 mg/m^2^, a dose that kills 95% of susceptible mosquitoes under laboratory conditions with 3 minutes exposure. Bednets were holed to mimic physical damage. The trial was conducted in three experimental huts from Pitoa, North-Cameroon where *Anopheles gambiae *displays metabolic resistance and cohabits with *An. funestus*.

**Results:**

Bifenthrin at 50 mg/m^2 ^significantly reduced anophelines' entry rate (>80%). This was not observed at 5 mg/m^2^. Both treatments increased exophily in *An. gambiae*, and to a lesser extent in *An. funestus*. With bifenthrin at high dosage, over 60% reduction in blood feeding and 75–90% mortality rates were observed in both vectors. Despite presence of holes, only a single *An. gambiae *and two *An. funestus *females were collected inside the treated net, and all were found dead. The same trends were observed with low dosage bifenthrin though in most cases, no significant difference was found with the untreated control net.

**Conclusion:**

Bifenthrin-impregnated bednets at 50 mg/m^2 ^were efficient in the reduction of human-vector contact in Pitoa. Considerable personal protection was gained against *An. funestus *and metabolic pyrethroid resistant *An. gambiae *populations.

## Background

Insecticide-treated bednets (ITNs) are being strongly promoted as a malaria control tool in Africa by the World Health Organization and other international agencies [1]. Their efficacy in reducing man-vector contact, malaria morbidity and mortality has been demonstrated in various epidemiological situations [2-5]. With current use of pyrethroids in agriculture and increasing scale of ITNs coverage, selective pressure for pyrethroid resistance in mosquitoes is expected to increase [6,7]. Resistance to pyrethroids has been reported in both *Anopheles gambiae *and *Anopheles funestus *in many malaria endemic countries in Africa [8-12], prompting for alternative products and strategies for malaria vector control [13,14]. Studies in experimental huts and village scale trials however, suggest that ITNs maintain their efficacy in areas with high frequency of *kdr *and insensitive acetylcholinesterase (*Ace.I*^*R*^) resistance genes in *An. gambiae *[15-19]. This should be investigated in areas where pyrethroid-resistance mechanisms other than these well-known target site mutations are incriminated [20,21]. Indeed, enzymes involved in insecticide detoxification may further jeopardize malaria vector control with insecticide-treated materials. This was recently exemplified in South Africa, where metabolic resistance to pyrethroids in *An. funestus *required a switch back from pyrethroids to DTT for house spraying to restore malaria control [9].

In this study, the efficacy of bednets impregnated with bifenthrin against natural populations of *An. gambiae *s.l. showing metabolic-based resistance was assessed in experimental huts. Artificially holed bednets were used in an attempt to mimic the damage that commonly occurs through domestic use. The effect of this treatment was assessed on the local mosquito populations, and the level of personal and mass protection expected from such control strategy was estimated.

## Methods

### Study area and mosquito populations

The trial was carried out in three experimental huts in Pitoa, North-Cameroon. Pitoa (9°21N; 13°31E), lies within the Soudanian climate domain with 700–1,000 mm annual rainfall. Malaria transmission in this area is seasonal, with sudden rise of new infections acquired during the rainy season (May-October). An. *gambiae *s.l. and *An. funestus *s.l. are the major malaria vectors. Laboratory tests revealed resistance to permethrin and deltamethrin but not to DDT in the *An. gambiae *s.l. population from Pitoa [12]. Biochemical analyses revealed increased oxidases and esterases activities in both *An. gambiae *and *An. arabiensis *(J. Etang, unpublished data).

### Experimental station

The field station is made of six standardized experimental huts situated near the "Mayo Pitoa", tributary of Benoue River. Each hut is 2.5 m long, 1.75 m wide and 2 m high [22]. The walls are made of concrete bricks, the floor of cement, with a corrugated iron roof. A plastic cover is stretched under the roofing sheets to facilitate hand catching of mosquitoes. Each hut is surrounded by a water-filled channel to prevent entry of ants. Entry of mosquitoes is only allowed through four window slits (1 cm wide) located on three sides of the hut, the slits being designed to prevent mosquitoes from escaping once they have entered the hut. Each hut is equipped with a veranda trap located on the fourth side, made of plastic sheeting and screening mesh.

### Bednets and insecticide

The nets were made of white 100-denier polyester (Vestergaard/Fransden). They measured 2 m in length, 1.2 m wide and 1.8 m high, and had a total surface area of 13.92 m^2^. To mimic domestic damage, 4 cm^2 ^regular holes were pierced so that the torn surface represented 0.8% of the total surface of the bednet. Bifenthrin, a non alpha-cyano pyrethroid insecticide, was used for this experiment as it has been successfully evaluated on netting materials under laboratory conditions, particularly against *knockdown-resistant *mosquitoes [23,24].

Nets were impregnated with bifenthrin (Talstar, 80 g/l SC) at the operational dose of 50 mg/m^2 ^[25,26] and at 5 mg/m^2^, a dose that kills 95% of susceptible mosquitoes under laboratory conditions with 3 minutes exposure [23,24]. Each net was dipped in an insecticide mixture and dried horizontally. Treated nets were randomly allocated to two experimental huts at the station. Another hut received a non-impregnated torn bednet and was used as a control.

### Mosquito collection and identification

Three volunteers from the locality of Pitoa were hired to sleep in each hut from 8.00 pm to 5.00 am. They gave informed consent and were trained in collecting mosquitoes. Ethical clearance was sought and granted from the National Ethics Committee of Cameroon and the trial was run for 120 nights from July to October 2004. To correct for possible differences in attractiveness to mosquitoes, the sleepers were rotated between huts every night. Mosquitoes were collected in the morning by hand catching under the bed net, inside the hut, and in the veranda. They were identified morphologically [27] and live females were held in netted plastic cups and supplied with sugar solution for 24 h before recording any delayed mortality. All anophelines specimens were stored individually in labelled tubes with desiccant for laboratory processing at OCEAC (Organisation de Coordination pour la lutte Contre les Endémies en Afrique Centrale) in Yaoundé. Differential identification of sibling species in the *An. gambiae *complex including simultaneous separation of M and S molecular forms within *An. gambiae *ss was carried out using PCR-RFLP [28].

### Data analysis

The effect of each treatment was assessed relative to the control arm in terms of deterrency (the expected reduction in the number of mosquitoes entering the hut), excito-repellency (the proportion of mosquitoes in the veranda traps), the proportion blood-fed and the proportion killed. Because of non-normality in the number of mosquitoes collected from each hut, the proportional data were analyzed using logistic regression (XLSTAT software). The significance of individuals coefficients estimated by the logistic regression model was tested using Wald statistic that follows Chi2 distribution (with df = 1).

## Results

### Dynamics of vector populations

**Figure 1:**
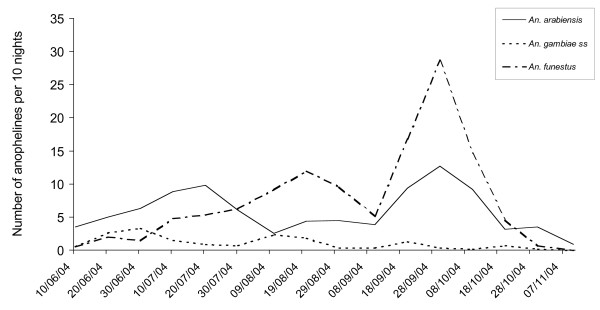
Dynamics of apparition of *An. gambiae *ss, *An. arabiensis *and *An. funestus *species in Pitoa, Northern Cameroon (results from the control hut hand catching).

Both *An. gambiae s.l*. and *An. funestus *female mosquitoes were collected in the huts. Results from the untreated hut showed a predominance of *An. gambiae s.l*. in the early rainy season and *An. funestus *gradually increased in abundance at the end of the survey (Fig. 1). Molecular identification of sibling species in the *An. gambiae *complex showed that the great majority of *An gambiae *s.l. were *An. arabiensis *(94/111 = 85% of the specimens identified), together with *An. gambiae *s.s. from the S molecular form (17/111 = 15%).

### Mosquito abundance

To assess any difference in attractiveness to mosquitoes, preliminary collections were carried out using untreated nets with the volunteers being rotated between huts on successive nights. These baseline measurements revealed no difference in attractiveness between huts (N = 298, F = 1.84, P = 0.11).

The total numbers of *An. gambiae *and *An. funestus *collected during the study are given in Table 1. A total of 730 mosquitoes were recorded over the 120 nights, of which 316 were *An. gambiae *and 344 were *An. funestus*. The remaining mosquitoes were composed of *Culex *sp (6.8%), *Mansonia *sp (1.4%) and *Aedes *sp (0.7%).

**Table 1:** *Anopheles gambiae *s.l. and *An. funestus *females collected in experimental huts in Pitoa.

		*An. gambiae *s.l.			*An. funestus*	
	Untreated	Bifenthrin 50 mg/m^2^	Bifenthrin 5 mg/m^2^	Untreated	Bifenthrin 50 mg/m^2^	Bifenthrin 5 mg/m^2^
Total number in hut	111^a^	21^b^	184^a^	122^a^	13^b^	209^a^
Exophily (%)	29.7^a^	61.9^b^	44.6^b^	20.5^a, c^	30.8^a, b, c^	15.8^c^
(95% CI)	(22.0–38.9)	(40.2–79.7)	(37.5–51.8)	(14.2–28.6)	(12.0–59.1)	11.4–21.4
Blood-fed (%)	91.0^a, b^	33.3^c^	84.2^a^	95.9^b^	38.5^c^	88.5^a^
(95% CI)	(84.1–95.1)	(16.8–55.3)	(78.2–88.8)	(90.5–98.3)	(17.0–65.6)	(83.4–92.2)
Overall Mortality (%)	9.9^a^	90.5^b^	20.7^c^	4.9^a^	76.9^b^	9.6^a^
(95% CI)	(5.6–17.0)	(68.9–97.6)	(15.4–27.1)	(2.2–10.5)	(47.8–92.4)	(6.3–14.4)
Immediate mortality (%)	3.6	71.4	6.5	0.0	76.9	1.9
Mortality unfed (%)	4.5	61.9	7.6	2.5	70.0	35.0

Numbers in the same row sharing a letter superscript do not differ significantly (*P *> 0.05), 95%CI: 95% Confidence Interval

### Entry rate

*An. gambiae *and *An. funestus *were present in about equal numbers in the control hut (Table 1). Bifenthrin at 50 mg/m^2 ^reduced the entry rate by 81.0% for *An. gambiae *and 89.3% for *An. funestus*. However, this deterrent effect was not detectable for either species with bifenthrin used at 5 mg/m^2^.

### Exophily

Bifenthrin at 50 mg/m^2 ^increased significantly the proportion of *An. gambiae *exiting into the veranda trap, up to 2.8 times the rate of natural exophily observed in the control hut (Wald Khi^2 ^= 7.4, *P *= 0.007). No induced exophily was noted for *An. funestus *(Wald Khi^2 ^= 0.7, *P *= 0.40). The same trend was observed with bifenthrin at 5 mg/m^2 ^which also significantly increased the rate of exophily for *An. gambiae *(Wald Khi^2 ^= 6.3, *P *= 0.01).

### Blood feeding

Bifenthrin at 50 mg/m^2 ^significantly reduced the rate of blood feeding for both *An. gambiae *(Wald Khi^2 ^= 27.9, *P *< 0.001) and *An. funestus *(Wald Khi^2 ^= 24.6, *P *< 0.001). Blood feeding inhibition was not significant with bifenthrin at 5 mg/m^2 ^in either vector species.

### Mortality

Less than 10.0% mortality was recorded in the control hut for either *An. gambiae *or *An. funestus *(Table 1). Bifenthrin at 50 mg/m^2 ^killed 90.5% of *An. gambiae *(Wald Khi ^2 ^= 30.4, *P *< 0.001), among which 79.0% were dead at the time of collection and 62.0% died unfed. At the same dosage, 77.0% of *An. funestus *were killed (Wald Khi^2 ^= 28.5, *P *< 0.001), all of them at the time of collection, and 70.0% died unfed. Bifenthrin at 5 mg/m^2 ^significantly increased the mortality rate of *An. gambiae *(Wald Khi^2 ^= 5.5, *P *= 0.02) but not of *An. funestus *(Wald Khi^2 ^= 2.2, *P *= 0.14).

### Chemical barrier

Compared with 36.0% of *An. gambiae *(40 out of 111) and 42.6% of *An. funestus *(52 out of 122) collected inside the untreated control net, only a single *An. gambiae *and two *An. funestus *females were collected inside the treated net at 50 mg/m^2^, and all were found unfed and dead.

Bifenthrin at 5 mg/m^2 ^decreased the number of *An. gambiae *entering the net but did not do so for *An. funestus*.

## Discussion

Insecticide Treated Nets (ITNs) not only provide personal protection; a high level of coverage in a population may benefit every individual in the community by contributing to an area-wide reduction in malaria vector populations [19]. The personal and mass effect protection that could be conferred by bifenthrin was estimated according to WHO guidelines [29]. The personal protection effect of a treatment relative to the control was estimated by the formula: 100 × (B_u_-B_t_)/B_u_, where B_u _is the total number of blood-fed mosquitoes in the untreated hut, and B_t _is the total number of blood-fed mosquitoes in the treated hut. The overall insecticidal effect of a treatment was estimated by the formula: 100 × (D_t_-D_u_)/E_u _where D_t _is the total number of mosquitoes dying in the treated hut, D_u _is the number dying in the untreated hut, and E_u _is the number of mosquitoes entering the untreated hut [29]. For both species, personal protection conferred by bifenthrin at 50 mg/m^2 ^was very high (more than 90%) while it was inexistent at 5 mg/m^2^. Conversely, the mass effect protection was very low, particularly at 50 mg/m^2 ^(Table 2).

**Table 2:** Comparison of personal and mass-effect protection conferred by torn bed nets impregnated with Bifrenthrin against pyrethroid resistant *An. gambiae *s.l. and *An. funestus *in Pitoa

Index	Mosquito Species	Bednet treatment	
		
		Bifenthrin (50 mg/m^2^)	Bifenthrin (5 mg/m^2^)
% personal protection*	*An. gambiae *s.l.	93.07	-53.47
	*An. funestus*	95.72	-58.11
% mass effect protection*	*An. gambiae *s.l.	7.21	24.32
	*An. funestus*	3.27	11.47

* See text and reference [29] for details on the formula use

The high level of personal protection conferred by bifenthrin at 50 mg/m^2 ^is a reflection of the significant level of blood feeding inhibition combined with the strong deterrent effect observed on the Pitoa mosquito populations (Table 1). Recent studies using the tunnel test technique in controlled condition also attributed this high inhibition of blood feeding to the irritant effect of bifenthrin [30], although it was found to be less irritant than other pyrethroids in laboratory conditions [23,24]. Hence, although the mortality rate was high with bifenthrin at 50 mg/m^2^, the deterrent effect greatly reduced the number of mosquitoes killed, thus resulting in a low mass protection effect.

Therefore, the deterrent effect, i.e. the reduction in the number of mosquitoes entering the hut, is not a reliable indicator of ITNs efficacy. Moreover, the values of this index vary considerably between experiments, e.g. from zero to 70.0% against *kdr-resistant An. gambiae*, even at the same study site in Côte d'Ivoire [25,26]. As a result, and acknowledging the absence of comparative studies involving enzyme-based pyrethroid resistance mechanisms, the high reduction of entry rate induced by bifenthrin in this study remains tentative and difficult to interpret. However, an obvious dose dependency of the deterrent effect was noted on both *An. gambiae *and *An. funestus *(Table 1).

For the same reason, the induced exophily does not permit an easy interpretation of the results. While exophily induced by bifenthrin is low in Côte d'Ivoire against *An. gambiae *(maximum value: 24.0%) [25], it was higher (Table 1) against the resistant *An. gambiae *population from Pitoa but less so against *An. funestus*. This is consistent with a former report demonstrating that *An. funestus *was 3 times less irritable as *An. gambiae *to DDT in North Cameroon [31]. It is tempting to speculate that such variability in the sensitivity of these vector species to the excito-repellent effect of insecticides applies with bifenthrin, a type I pyrethroid with the same neuro-physiological mode of action than DDT. Furthermore, the great majority of *An. gambiae s.l*. identified in Pitoa was constituted by *An. arabiensis*, which might be differentially affected by the excito-repellent effect of bifenthrin than its sibling species. This might reflect species-specific variability in behavioural traits linked to host-seeking, biting and/or feeding behaviour and suggests that mosquito behaviour might modulate to a considerable extent the efficacy of insecticides and ITNs in natural conditions.

Almost all the *An. arabiensis *and *An. funestus *fed in the presence of the untreated net but high dose of bifenthrin markedly reduced these rates in both vectors (Table 1). In addition, a great proportion of *An. arabiensis *and *An. funestus *entering the hut with a net treated at 50 mg/m^2 ^were found dead at the time of collection, in similar proportions to what has been reported with bifenthrin [25,26], but in higher proportions than what was observed with deltamethrin [16]. As such, treatment with the high dose bifenthrin restored the protectiveness of torn nets against *An. arabiensis *and *An. funestus*, as has been demonstrated with various pyrethroid treatments and against various mosquito species and populations [19,32-35]. Both blood-feeding inhibition and mortality rates appeared to be dosage-dependant, inasmuch as the effect of bifenthrin at 5 mg/m^2 ^on these indicators was low and, in most case, non-significant (Table 1). Further tests with intermediate concentration would however be needed to confirm a dosage trend.

Moreover, no data are currently available on the susceptibility of *An. funestus *to insecticides in this area and this needs to be monitored, acknowledging enzyme-based insecticide resistance was recently detected in populations from South Africa [9]. Additionally, although resistance to permethrin and deltamethrin was demonstrated in *An. gambiae s.l*. from Pitoa [12], susceptibility to bifenthrin has not yet been evaluated and cross-resistance between these compounds should not be assumed due to high substrate specificity and selectivity of the enzymes involved in pyrethroids detoxification pathways [36,37].

## Conclusion

The results presented above confirm that bifenthrin at 50 mg/m^2 ^is a suitable dosage that could be recommended for mosquito net impregnation, even in areas of vector resistance to commonly used pyrethroids (permethrin and deltamethrin) and regardless of the mechanisms involved (*kdr *or enzyme-based). Bifenthrin impregnated bed nets offer considerable personal protection, even in the presence of holes in the net. However, its strong deterrent effect might jeopardize any area-wide impact on vector populations, as should be expected from large scale intervention programs.

## Authors' contributions

MC carried out the field evaluation of nets, analysed and interpreted data and drafted the manuscript. FS participated in the conception of the study and coordinated its implementation in the fields, and substantially helped draft the manuscript and data interpretation. FC participated in the conception of the study and its design and helped in data analysis and interpretation. JE participated in data analysis and interpretation. FD set up the field station in Pitoa & JMH planned the study and its design, participated in data analysis and interpretation, and helped draft the manuscript.
